# A Neural Algorithm for the Detection and Correction of Anomalies: Application to the Landing of an Airplane

**DOI:** 10.3390/s22062334

**Published:** 2022-03-17

**Authors:** Angel Mur, Louise Travé-Massuyès, Elodie Chanthery, Renaud Pons, Pauline Ribot

**Affiliations:** LAAS-CNRS, Université de Toulouse, 7 Av. du Colonel Roche, 31400 Toulouse, France; louise@laas.fr (L.T.-M.); elodie.chanthery@laas.fr (E.C.); renaud.pons@laas.fr (R.P.); pauline.ribot@laas.fr (P.R.)

**Keywords:** anomaly detection, anomaly correction, deep learning, airplane landing

## Abstract

The location of the plane is key during the landing operation. A set of sensors provides data to get the best estimation of plane localization. However, data can contain anomalies. To guarantee correct behavior of the sensors, anomalies must be detected. Then, either the faulty sensor is isolated or the detected anomaly is filtered. This article presents a new neural algorithm for the detection and correction of anomalies named NADCA. This algorithm uses a compact deep learning prediction model and has been evaluated using real and simulated anomalies in real landing signals. NADCA detects and corrects both fast-changing and slow-moving anomalies; it is robust regardless of the degree of oscillation of the signals and sensors with abnormal behavior do not need to be isolated. NADCA can detect and correct anomalies in real time regardless of sensor accuracy. Likewise, NADCA can deal with simultaneous anomalies in different sensors and avoid possible problems of coupling between signals. From a technical point of view, NADCA uses a new prediction method and a new approach to obtain a smoothed signal in real time. NADCA has been developed to detect and correct anomalies during the landing of an airplane, hence improving the information presented to the pilot. Nevertheless, NADCA is a general-purpose algorithm that could be useful in other contexts. NADCA evaluation has given an average *F*-score value of 0.97 for anomaly detection and an average root mean square error (RMSE) value of 2.10 for anomaly correction.

## 1. Introduction

Anomaly detection is about finding patterns that do not adhere to what is considered normal behavior [1]. Abnormal events are a major problem as people’s lives can be at risk and companies as well as public institutions can suffer serious losses.

Fraudulent activity in the banking sector, deforestation in the environmental sector, cancer in the healthcare sector, fake news in the social media sector, hacker attacks in cybersecurity, malfunctions in the manufacturing sector, traffic jams in the transportation sector, etc. are some examples of anomalies. Some examples of anomaly detection in different fields are presented in [2,3,4,5,6].

Commercial aircraft flights are a good example where anomaly detection is very important. Although fault tolerant architectures are in place, anomaly detection is paramount to passivate faulty components. A faulty actuator can be switched to its sane redundant counterpart. A faulty sensor can be put aside from the data fusion process [7]. In particular, the location of an airplane is an essential piece of information during the landing process. It is obtained from a set of sensors that present redundancies and whose values are fused. Thus, each sensor involved in the data fusion must provide measures without anomalies.

Normally, the set of sensors consists of a global positioning system (GPS), an inertial reference system (IRS), an instrument landing system (ILS), and a radio-altimeter (RA). Typically, these sensors work properly with a specific accuracy and specific fusion techniques are applied to get a good estimate of the airplane’s location [7].

However, sensors can provide data with anomalies. Anomaly detection methods can be applied to guarantee optimal quality of measures. When an anomaly is detected, either the anomalous sensor is isolated or the detected anomaly is filtered.

This article presents a new algorithm named NADCA (Neural Algorithm for the Detection and Correction of Anomalies) to detect and correct anomalies in time series. This algorithm is a general-purpose algorithm, but it has been developed in the framework of a project in the field of aeronautics to detect and correct sensor anomalies during airplane landing.

NADCA uses a predictive model based on deep learning. More precisely, NADCA is based on a recurrent neural network (RNN) called Long Short-Term Memory (LSTM) [8].

Deep learning has been used with success for classification and prediction purposes [9]. In particular, different NN architectures have been successfully leveraged for time series analysis [9]. Deep learning has the ability to automatically discover complex features without having any domain knowledge. Consequently, NN is a good platform to solve the time series anomaly detection problem.

LSTM is a good choice for the prediction task of time series because it can deal with chronologically ordered sequences and can track long-term dependencies in these sequences. Like most NN-based algorithms, LSTM relies on the assumption that training and test data share similar statistics.

In [10], various deep learning models for anomaly detection, including prediction methods, are investigated. Their suitability for a given data set is also analyzed. A more recent review about deep anomaly detection is provided in [11]. This work reviews 12 diverse modeling perspectives on leveraging deep learning techniques for the detection of anomalies. It also discusses how these methods address some notorious anomaly detection challenges to demonstrate the importance of deep anomaly detection.

An anomaly detection technique based on LSTM is proposed in [12]. The model is trained using normal data. Then, the prediction error distribution between measure and prediction is computed. An error threshold allows to decide when the time series has a normal or anomalous behavior. An LSTM-based encoder-decoder for multi-sensor anomaly detection is presented in [13]. Another deep learning method to detect anomalies in time series combining wavelet transform and NNs is presented in [14]. In [15], LSTM is used for detecting anomalies in flight data. A set of eleven canonical anomalies is tested.

A more recent work uses convolutional neural networks (*CNNs*) to detect anomalies [16]. This approach allows to obtain a model that generalizes well without using a large number of examples during the learning process. This is possible as CNNs achieve a good parameter selection.

Autoencoders are NNs that learn to copy their input to their output. In [17], autoencoders are also used to detect anomalies.

Unlike the above deep learning methods, NADCA uses differences between consecutive measures to train a model. The model predicts a difference in each iteration. This difference added to the corresponding measure produces the prediction of the next measure. This approach is advantageous because the prediction does not depend on the accuracy of the sensor and reduces non-stationary aspects of the original time series. Moreover, the prediction of a single difference does not require a significant number of previous measurements. This fact reduces the necessary number of examples during training.

Another original aspect resides in the design of NADCA. NADCA allows data to be processed in a general way regardless of the degree of oscillation present in the sensor data. That is interesting because NADCA only predicts a sample and uses a small number of measures at each iteration.

The criterion for deciding whether a measure is an anomaly or not is also different. The algorithm compares a prediction with the corresponding measure and uses a threshold (*U*) to decide. The threshold can be fixed or adaptive depending on the nature of the data. The prediction is always obtained from a smooth signal, i.e., the signal is smoothed when it shows oscillations. A signal without oscillations is defined as a signal whose smoothed signal is the same as the original signal (more explanations in Section 2.6).

Predicting from a smooth signal makes the prediction error small and less than a constant. This means that the algorithm is robust for the detection and correction of anomalies regardless of the degree of oscillation of the signal.

When the signal has no oscillations, the threshold *U* is the maximum prediction error. When the signal has oscillations, *U* is the maximum distance among the samples between the smoothed signal and the raw values. In both cases, *U* is determined using a set of signals without anomalies. This approach detects both fast-changing and slow-moving anomalies.

Regarding anomaly detection in sensors during landing, the work of [18] stands out. In that thesis, the author provides a comparative analysis of several existing machine learning techniques to detect anomalies. The faulty sensor is isolated once the anomaly has been detected. The simulation of the sensors during landing is another important aspect of this work. In this way, data are easily obtained to test the algorithms.

Beyond the analysis of [18], an original aspect of our work is the use of an algorithm that allows the detection of anomalies together with their correction. Note that the NADCA algorithm is especially designed to deal with anomalies during the landing phase where airplanes normally do not have abrupt trajectory changes. During a sudden change of trajectory, NADCA could detect anomalies in all the sensors.

A more recent paper studies the stability of aircraft lateral movement during the ILS approach [19]. To estimate the lateral stability index, a gated recurrent unit (GRU) [20] is used where GRU is a simplified version of LSTM.

Concerning landing data, NADCA analyzes anomalies according to the X, Y, and Z axes of the runway reference system. The values of the sensors according to these reference axes can be coupled. When this occurs, the origin of the anomaly is unclear. However, the existence of coupling is not a problem for NADCA. NADCA detects and corrects the anomalies following the order X, Y, and Z. If an anomaly appears in any sensor coordinate, it is corrected before analyzing the next coordinate, since the latter can be a function of the first coordinate.

Each coordinate can be represented by a multichannel signal (a channel per sensor). NADCA uses a unique predictive model per coordinate. The prediction is carried out in a compact way, encouraging the sensors to help each other. The prediction on each sensor is used to detect and correct each anomaly. Ref. [21] also considers multichannel signals compactly but only to detect anomalies. It does not perform a correction of the anomaly, and it does not prevent possible coupling effects. In contrast to NADCA, the algorithm is unsupervised and does not need training.

From a technical point of view, NADCA has two important innovations. As explained, the algorithm compares a prediction with the corresponding measure and uses *U* to decide. This is also the basic behavior of an algorithm to detect anomalies using a predictive model. Anomalies that change abruptly, that is, in the time interval between two consecutive samples, are easily detected. However, there are many anomalies that vary more slowly. When this happens, anomaly detection algorithms that use this basic behavior fail. This occurs since the prediction is calculated from the closest previous measurement. NADCA solves this problem using a new strategy to calculate this prediction. It can even detect and correct drift anomalies. On the other hand, NADCA can also work with signals regardless of whether the signal has oscillations or not. A similar algorithm is applied for both types of signals. However, for signals with oscillations, an additional step is necessary to obtain a smoothed signal. The smoothed signal is created in real time and this is also a novel aspect.

To summarize, the advantages of our approach are as follows: it is suitable for working with multiple time series, it provides a compact model for all sensors, detection and correction of any anomaly is done at the same time, it is robust regardless of the degree of oscillation of the signals, it detects both fast-changing and slow-moving anomalies, it only needs a small number of measures at each iteration because it predicts one sample, the characteristics of the anomaly (e.g., type, duration, etc.) can be selected and sensor behavior can be analyzed, sensors with abnormal behavior do not need to be isolated because NADCA produces corrected values, it does not depend on the accuracy of the sensor, it can cope with simultaneous anomalies on different sensors, it can be implemented in real time, and it can detect the origin of any anomaly avoiding the coupling problem.

As far as we know, there is no other algorithm capable of detecting and correcting anomalies with all these advantages, especially when the algorithm is applied during the landing process.

This article is organized as follows. Section 2 reviews some basic concepts referring to the aircraft landing phase and to the neuronal tools used by NADCA. Section 3 describes the algorithm NADCA. Section 4 explains some elements of NADCA using real landings while Section 5 shows some examples of anomaly detection and correction using NADCA. Section 6 discusses the methodology and results. Finally, Section 7 concludes the article.

## 2. Background

This section reviews some important concepts for understanding NADCA, as well as for understanding the aircraft landing application.

### 2.1. Admissible Work Interval for Detecting and Correcting Anomalies during Landing

**Figure 1:**
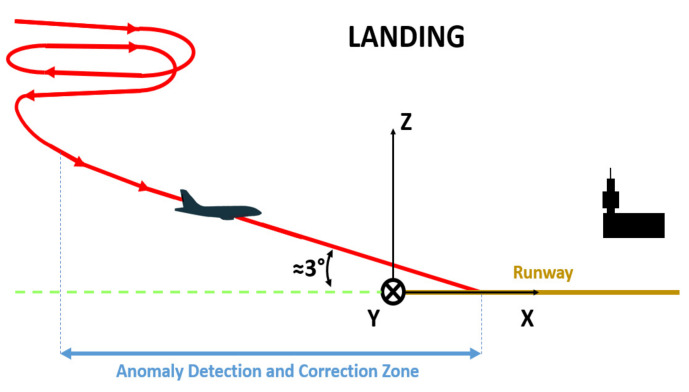
Anomaly detection and correction zone during the landing of an airplane.

A coordinate system is placed at the origin of the runway (see Figure 1). The plane begins to land when it is almost aligned with the X axis of the runway. The landing ends when the plane makes contact with the runway. The NADCA algorithm works in that interval.

### 2.2. Sequence Prediction and Time Series

Supervised machine learning algorithms use a set of samples for the training process. Each sample is an observation or measure.

Machine learning algorithms can be used for sequence prediction. Sequence prediction involves predicting the next value for a given input sequence. In this case, the set of samples is different because a sequence describes a set of ordered measures (for example, measures ordered chronologically, i.e., times series). Consequently, the order of the samples used in the algorithms must be respected.

In this article, time series from a set of sensors are used. The concepts of time series and signal are used indistinctly. Predictions in times series are made with the help of a LSTM network.

### 2.3. LSTM Network

An LSTM network is a kind of RNN [9]. It attempts to model sequence-dependent behavior by feeding back the output of a NN layer at time *t* to the input of the same NN layer at time *t* + 1. LSTM propagates the information learned at a time *t* to the future. In general, a classic RNN likes to remember everything. By contrast, LSTM saves relevant information and forgets information that is not important.

LSTM architectures are not unique. Depending on the type of problem, some architectures perform better than others. Some architectures are as follows: vanilla, stacked, CNN, encoder-decoder, etc. [22,23]. We selected a Stacked architecture in which LSTM layers are stacked one on top of another into deep networks.

An LSTM network was used to create the predictive model of NADCA. This supervised algorithm predicts acceptably if it has been trained with a significant number of examples. Predictions are robust when the predictive model is used in time series with no oscillations.

### 2.4. Sensors, Signals, Location, and Coupling

During a landing, the complete set of signals with respect to the runway reference can be described by three multichannel signals: [*X^GPS^*, *X^IRS^*] for the X coordinate, [*Y^ILS^*, *Y^GPS^*, *Y^IRS^*] for the *Y* coordinate, and [*Z^ILS^*, *Z^RA^*, *Z^GPS^*, *Z^IRS^*] for the *Z* coordinate. Each signal is denoted by the “*Coordinate*^Sensor^” symbol.

The airplane’s GPS provides latitude, longitude, and altitude. These values represent the position of the airplane in geodesic coordinates (WGS84). The airplane location with respect to the runway (*X^GPS^*, *Y^GPS^*, *Z^GPS^*) can be calculated by means of a coordinate system conversion. In a similar way, the airplane location provided by the IRS with respect to the runway (*X^IRS^*, *Y^IRS^*, *Z^IRS^*) can be calculated.

The radio altimeter measures the aircraft altitude (*H^RA^*), i.e., the vertical distance between the aircraft and the ground. In order to get *Z^RA^*, one must apply a correction with respect to the relief under the aircraft, using a terrain database:

(1) $$Z^{RA}=H^{RA}+H_{terrain}$$

where *H_terrain_* is the altitude of the terrain with respect to the runway threshold. The *H_terrain_* value can be obtained using the *X^GPS^* or *X^IRS^* values.

The ILS is a ground-based system that emits signals along the vertical and lateral axis so that the aircraft can follow a line of reference named the localizer (LOC) in the lateral axis and the glideslope (GS) on the vertical axis. The ILS can be manipulated to obtain the airplane’s position coordinates with respect to the runway (*Y^ILS^*, *Z^ILS^*). These values can be calculated using Equations (2) and (3). These equations provide a good approximation to the real values [18].

(2) $$Y^{ILS}=\frac{\sigma_{LOC\;}\times s\times L-X}{L}$$

where *L* is the runway length (usually 3500 m), *s* is the *LOC* sensitivity (usually 0.7 m/μA) and $\sigma_{LOC\;}$ is the *LOC* deviation in μA. The *X* value can be obtained using the *X^GPS^* or *X^IRS^* values.

(3) $$Z^{ILS}=\left|X+300\right|\times \tan\left(GPA+\rho_{GS}\right)$$

where *GPA* is the angle of reference (3°) and $\rho_{GS}$ is the noise of the *GS*. The *X* value can be obtained using the *X^GPS^* or *X^IRS^* values.

The GPS and IRS coordinates do not depend on the coordinates of other sensors. However, *Z^RA^*, *Y^ILS^*, and *Z^ILS^* depend on the GPS or IRS. NADCA avoids this coupling because it detects and corrects anomalies following the order *X*, *Y*, and *Z*. An *X^GPS^* anomaly (or *X^IRS^* anomaly) is detected and corrected before the corresponding values are used to calculate *Z^RA^*, *Y^ILS^*, and *Z^ILS^*.

**Figure 2:**
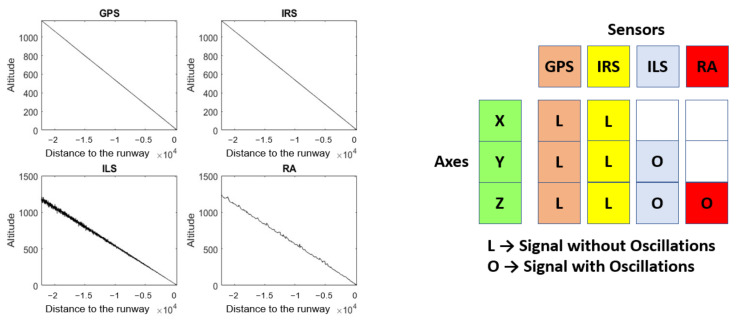
Example of simulated time series of the *Z* coordinate during the landing process: *Z^GPS^*, *Z^IRS^*, *Z^ILS^*, and *Z^RA^*. On the right side, a table relates each sensor to each coordinate. The sensor coordinate cell shows whether or not the signal has oscillations. There is no signal if the cell is empty.

Figure 2 shows the *Z* coordinate of four simulated time series (*Z^GPS^*, *Z^IRS^*, *Z^ILS^*, and *Z^RA^*) during the landing process. Unlike the *Z* coordinate of GPS and IRS, the *Z* coordinate of ILS and RA is a signal with oscillations. A table, to the right of Figure 2, crosses the coordinates (according to the runway reference system) and signal for each sensor. In addition, the sensor coordinate cell indicates whether or not the signal has oscillations.

NADCA acts on each coordinate independently and takes into account whether the signal has oscillations or not.

### 2.5. Predictive Models

NADCA works on each *X*, *Y*, and *Z* axis independently. Therefore, there are three prediction models (*PM^X^*, *PM^Y^*, and *PM^Z^*), one for each axis. Each predictive model only works with signals without oscillations. This means that for ILS and RA signals, a smoothed signal is constructed in real time before being used by the predictive model. A letter *L* is used to denote the corresponding smoothed signals. Working with smoothed signals guarantees a low and stable prediction error.

**Figure 3:**
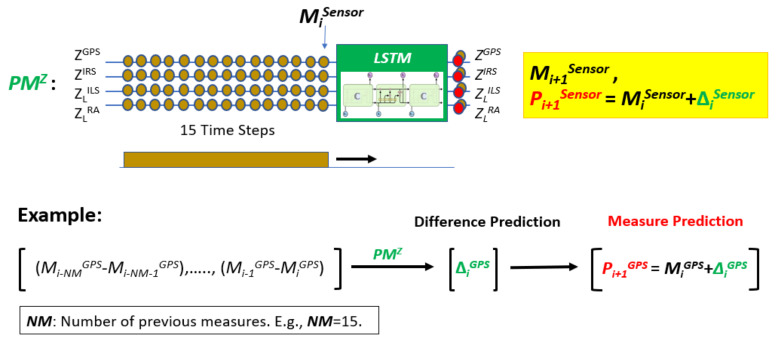
*PM^Z^* prediction. In the lower part, an example of measure prediction for the GPS is explained.

Figure 3 shows a predictive model for the Z axis denoted *PM^Z^*. It predicts using the multichannel signal (*Z_L_^ILS^*, *Z_L_^RA^*, *Z^GPS^*, *Z^IRS^*) where *Z_L_^ILS^* and *Z_L_^RA^* are the corresponding smooth signals of *Z^ILS^* and *Z^RA^*. *PM^Z^* predicts a difference of consecutive measurements from a set of differences obtained from some previous measurements. In this example, the predictive model takes 15 measurements, or 14 differences for each sensor up to sample *i*. Then, an LSTM compact architecture predicts a difference of measurements at time *i* + 1 for each sensor. The prediction of the measurement at time *i* + 1 ($P_{i+1}^{Sensor}$) is equal to the predicted difference ($\Delta_{i}^{Sensor}$) plus the measurement at time *i* ($M_{i}^{Sensor}$). Figure 3 also shows the difference prediction and measure prediction for GPS where the letter *Z* is not used for simplicity.

Likewise, NADCA uses a *PM^Y^* that acts on [*Y_L_ ^ILS^*, *Y ^GPS^*, *Y ^IRS^*] and a *PM^X^* that acts on [*X^GPS^*, *X^IRS^*]. The *PM^Z^* works with an LSTM network whose main architecture has 3 stacked layers with 300 cells per layer. Similar architectures are used for *PM^Y^* and *PM^X^*.

### 2.6. Smoothing Data with the Savitzky–Golay Filter

The Savitzky–Golay filter (*SG*) [24] is a particular type of low-pass filter, well adapted for data smoothing.

The *SG* filter removes high frequency noise from data. It has the advantage of preserving the original shape and features of the signal better than other types of filtering approaches, such as moving average techniques. The main idea behind this approach is to make for each point a least-square fit with a polynomial of high order over an odd-sized window centered at the point.

This filter is useful for obtaining a smoothed signal from a signal with oscillations and is used for ILS and RA signals in our approach.

## 3. Neural Algorithm for the Detection and Correction of Anomalies (NADCA)

The main elements of NADCA are the following:

− Sensor measurements (... *M_i_*_−1_, *M_i_*, *M_i_*_+1_).
− A reference *P_i_*_+1_ using a predictive model *PM*.
− A threshold *U*$\in {\mathbb{R}}^{+}$.

**Figure 4:**
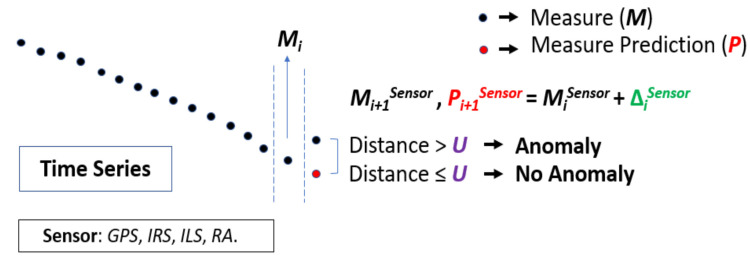
Main elements and basic behavior of NADCA. The red dot at time *i* + 1 is the measure prediction.

The basic version of NADCA (see Figure 4), named NADCA-B, is summarized in Algorithm 1 as follows:

**Algorithm 1:** NADCA-B algorithm.If the distance (absolute difference) between *M_i_*_+1_ and *P_i_*_+1_ is *> U* then “Anomaly”  If “Anomaly” then “Anomaly Correction” using predictions. else “No Anomaly”

In general, sensor data are non-stationary during landing. To work with stationary data, differences between consecutive data values are calculated. In this way, the predictive model predicts a difference Δ_i_ at each iteration *i* instead of a raw measure value. This prediction is hence independent of the sensor accuracy.

The difference $\Delta_{i}$ is added to the measure $M_{i}$ to predict the measure at time *i* + 1. The closer the value of this prediction $P_{i+1}$ is to the measure $M_{i+1}$, the better the prediction. The predictive model predicts a difference Δ_i_ from a set of previous differences *PD* = [$D_{i-ND}$, …, $D_{i-1}$] where *ND* is the number of differences used and $D_{i-1}$=$M_{i}$ − $M_{i-1}$. The number of previous measures is denoted *NM*. For example, if *NM* = 15, then *ND* = 14.

NADCA-B is simple but not always effective in detecting and correcting any type of anomaly. The maximum prediction error between *P_i_*_+1_ and *M_i_*_+1_ must be small and less than a constant, but NADCA-B does not always produce such prediction error. To optimally detect and correct any anomaly, a generalization of NADCA-B is necessary. This generalization is explained according to how NADCA-B is used in signals without oscillations (NADCA-L) or in signals with oscillations (NADCA-O).

### 3.1. NADCA-L: Generalization of NADCA-B for Signals without Oscillations

**Figure 5:**
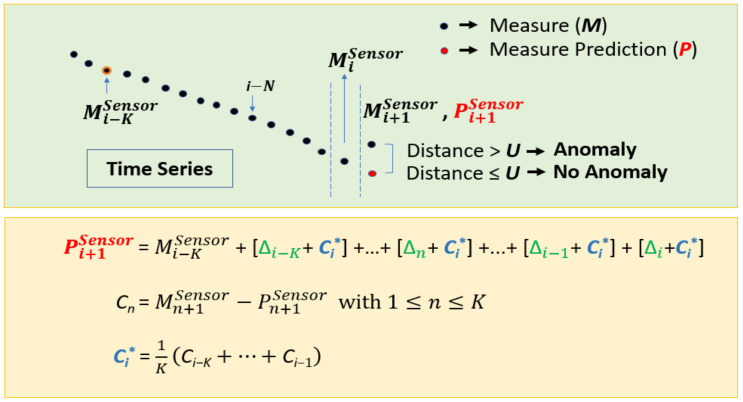
NADCA-L: Generalization of NADCA-B for anomaly detection and correction in signals without oscillations.

Figure 5 explains in detail how *NADCA-L* detects and corrects anomalies using a generalization of *NADCA-B*.

This generalization means that the prediction at *i* + 1 can be approximated in different ways.

If $P_{i+1}^{Sensor}=M_{i}^{Sensor}+\Delta_{i}$ is a good approximation of the real measure at time i+1, the following approximation $P_{i+1}^{Sensor}=M_{i-1}^{Sensor}+\Delta_{i-1}+\Delta_{i}$ also offers a small prediction error. In general, $P_{i+1}^{Sensor}=M_{i-K}^{Sensor}+\Delta_{i-K}+\cdots +\Delta_{i}$ where *K* is a positive integer indexing an initial measure $\left(IM=M_{i-K}^{Sensor}\right)$. A more precise equation is as follows:

(4) $$P_{i+1}^{Sensor}=IM+{\left(\Delta C\right)}_{i-K}+\cdots +{\left(\Delta C\right)}_{i-n}+\cdots +{\left(\Delta C\right)}_{i-1}+(\Delta_{i}+C_{i}^{*})$$

where ${\left(\Delta C\right)}_{i-n}=\Delta_{i-n}+C_{i}^{*}$, $C_{i}^{*}=\frac{1}{K}\left(C_{i-K}+\cdots +C_{i-1}\right)$, $C_{n}=M_{n+1}^{Sensor}-P_{n+1}^{Sensor}$ is a prediction error for $\Delta_{n}$ and *n* is an integer.

The $C_{i}^{*}$ parameter represents a correction by the average of the prediction error on the *K* last time points. It works well for fast-changing anomalies (e.g., noise). However, slow-moving anomalies such as drift might not be well detected.

For a potential slow-moving anomaly, $C_{i}^{*}$ is increased as *i* increases. The following equation shows that a drift-like anomaly starts at sample *i-N* if:

(5) $$\left\{C_{i-nc}^{*}-C_{i-\left(nc-1\right)}^{*}>0\right\}\;$$

where 1≤nc≤N and *N* < *K*. The value of *N* is fixed, e.g., *N* = 15. A new $C_{i}^{**}=C_{i-N}^{*}$ is selected and is used to detect a potential slow-moving anomaly.

In general, $C_{i}^{**}$ is close to or equal to $C_{i}^{*}$ when there is no anomaly or when there is a fast-changing anomaly. For a slow-moving anomaly, the value of $C_{i}^{**}$ is fixed using Equation (5) to detect the anomaly in the following iterations. Equation (4) allows to calculate $P_{i+1}$ (for simplicity, the exponent “sensor” has been omitted) using $C_{i}^{*}$. A new $P_{i+1}^{**}$ could also be obtained using $C_{i}^{**}$ instead of $C_{i}^{*}$ in (4).

If the following condition is true

(6) $$|M_{i+1}-P_{i+1}|>U$$

then there is an anomaly (mainly a fast-moving anomaly). However, a slow-moving anomaly is detected if

(7) $$\left|M_{i+1}-P_{i+1}\left|+\right|P_{i+1}-P_{i+1}^{**}\right|>U,\;\mathrm{that}\;\mathrm{is},\;\left|M_{i+1}-P_{i+1}\left|+\right|C_{i}^{*}-C_{i}^{**}\right|\;>\;U.$$

Equation (7) is necessary since $C_{i}^{**}$ and $C_{i}^{*}$ can move away at some point and however, this does not mean that a slow-moving anomaly is starting.

$P_{i+1}^{Sensor}$ is a reference for NADCA-L at each iteration. The set of all predicted values {$P_{i+1}^{Sensor}$} can be denoted by *Ref^Sensor^*.

In addition, NADCA-L also uses Equation (4) for correcting an anomaly in real time once it has been detected. If the anomaly has a short duration, Equation (4) is good enough to make the correction. For a long duration anomaly, a small deviation might appear. In this case, given an anomaly starting at sample *i*, the following equation could be used to improve quality of the correction:

(8) $$P_{j}=P_{j}+\;\alpha \;\times M$$

where *j* is a sample within the anomaly and *M* = *j* − *i*. The parameter α can be determined experimentally (see Section 5.1).

The NADCA-L method is summarized in Algorithm 2 as follows:

**Algorithm 2:** NADCA-L algorithm.Given a sample *i*, *U*, a set of *NM* measures [$M_{i-NM-1}$…$M_{i}$], a set of *K* predictions [$\Delta_{i-K}$…$\Delta_{i-1}$] and prediction errors [$C_{i-K}$…$C_{i-1}$] for the set of measures [$M_{i-K}$…$M_{i-1}$] and $M_{i+1}$: 1. Calculate the set of differences *PD* using the *NM* measures. 2. Calculate $\Delta_{i}$ using *PM* and *PD*. 3. Calculate $C_{i}^{*}$ and $C_{i}^{**}$. 4. Calculate $P_{i+1}^{Sensor}$ and $P_{i+1}^{Sensor}{}^{**}$ using (4). 5. Calculate $dis1=|M_{i+1}^{Sensor}-P_{i+1}^{Sensor}|$ and $dis2=\left|M_{i+1}^{Sensor}-P_{i+1}^{Sensor}\left|+\right|C_{i}^{*}-C_{i}^{**}\right|$ 6. If dis1 ≤ U and dis2 ≤U then “No anomaly” at i + 1. Save ($\Delta_{i}$, $C_{i}$) for the next iteration. Updating K ←K+1 allows the same *IM* to be used for the next iteration. 7. If dis1>U then “fast-changing anomaly” at *i* + 1. Correct the anomaly at *i* + 1 changing $M_{i+1}$ to $P_{i+1}$. Save ($\Delta_{i}$, $C_{i}$) for the next iteration. Updating *K* ←*K*+1 allows the same *IM* to be used for the next iteration. 8. If dis2>U and dis1<U then “slow-moving anomaly” at *i* + 1. Correct the anomaly at *i* + 1 changing $M_{i+1}$ to $P_{i+1}$. Save ($\Delta_{i}$, $C_{i}$) for the next iteration. Updating *K* ←*K*+1 allows the same *IM* to be used for the next iteration.

**Figure 6:**
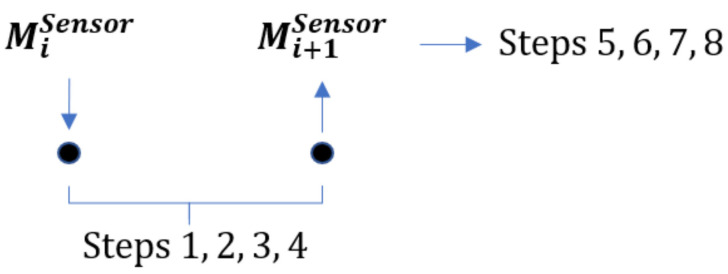
Steps of NADCA-L.

NADCA-L works in real time. This means that steps 1–4 described above are calculated during the time difference between two consecutive samples (sampling period). Once $M_{i+1}^{Sensor}$ is known, steps 5–8 allow to decide if there is anomaly or not (see Figure 6).

### 3.2. NADCA-O: Generalization of NADCA-B for Signals with Oscillations

**Figure 7:**
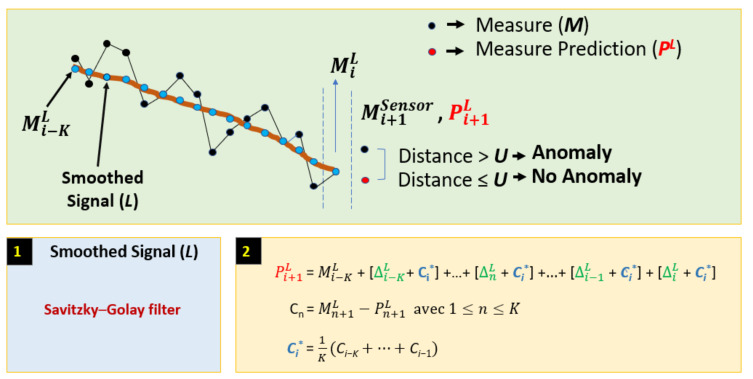
NADCA-O: Generalization of NADCA for anomaly detection and correction in signals with oscillations.

Figure 7 explains in detail how NADCA-O detects and corrects anomalies in signals with oscillations.

In general, the predictive model applied to the raw data of a non-stationary oscillating signal does not have a small prediction error less than a constant. This characteristic is not good for detecting and correcting anomalies in a robust way. One solution is to find a smooth signal (*L*) from the raw data. Each prediction on this smoothed signal constitutes a reference to determine if there is an anomaly or not. As the smooth signal does not present oscillations, the prediction error is small and less than a constant (e.g., in Section 4.3.1, prediction errors are calculated. GPS and IRS envelopes are constant lines).

NADCA-O contains two steps: the determination of *L* in real time and the NADCA-L algorithm.

A SG filter is used to determine *L* in real time. The SG filter is a general approach where the smooth signal depends only on the sensor data.

Given a set of *NT* measures [$M_{i-NT-1}$…$M_{i}$], the SG filter can be applied to obtain the corresponding smooth measures [$M_{i-NT-1}^{L}$ … $M_{i}^{L}$]. Typically, this process takes place offline. The *SG* filter uses a sliding window of, for example, about *NS* = 100 measurements (NS≤NT).

We want to apply the SG filter on a signal in real time where in the first iteration there are only *NM* samples (e.g., *NM* = 15) and for the next iterations, one sample per iteration is added. In general, the *NM* value is inferior to *NS*. To apply the SG filter in real time where only *NM* measurements are available in the first iteration, two changes are required. First, synthetic samples are added by repeating the set [$M_{1}$…$M_{NM}$] until the selected *NS* value is reached. After some iterations, synthetic samples are not necessary, and for each sample *i*, the measures [$M_{i-NM-1}$…$M_{i}$] are the last measures of the set [$M_{i-NT-1}$…$M_{i}$]. Second, at *i*, the *SG* filter is applied using the set of measures [$M_{i-NT}^{L}$ … $M_{i-1}^{L}$;$\;M_{i}$] to get $M_{i}^{L}$. Consequently, with both changes, the real-time SG filter result is of good quality, similar to an offline result.

With NADCA-O, the threshold *U* is the maximum distance between the prediction of the smooth signal $P_{i+1}^{L}$ and the measurement of the original signal $M_{i+1}^{Sensor}$. The value of *U* is determined by selecting the maximum value for each sample from a set of normal landings. In general, *U* is not constant for all samples.

The NADCA-O is summarized in Algorithm 3 as follows:

**Algorithm 3:** NADCA-O algorithm.Given a sample *i*, *U*, *NT*, a set of measures [$M_{i-NM-1}$…$M_{i}$] and $M_{i+1}$: 1. Calculate [$M_{i-NT-1}^{L}$ … $M_{i}^{L}$] using *NT* measures and the *SG* filter. If [$M_{i-NT-1}^{L}$ … $M_{i-1}^{L}$] is known, use the *SG* filter over the set [$M_{i-NT-1}^{L}$ … $M_{i-1}^{L}$,$\;M_{i}$]. 2. Calculate the set of differences *PD* using a set of *NM* measures [$M_{i-NM-1}^{L}$ … $M_{i}^{L}$]. 3. Calculate $\Delta_{i}^{L}$ using *PM* and *PD*. 4. Use *NADCA-L* where $P_{i+1}^{L}$ replaces $P_{i+1}^{Sensor}$.

NADCA-O works in real time. It means that steps 1–3 described above are calculated during the time difference between two consecutive samples. Once $M_{i+1}^{Sensor}$ is known, step 4 allows to decide if there is anomaly or not.

## 4. NADCA for Real Landings

A set of 36 landings from the same airport was selected. Each landing had the following signals: [*Z^ILS^*, *Z^RA^*, *Z^GPS^*, *Z^IRS^*] for the *Z* coordinate, [*Y^ILS^*, *Y^GPS^*, *Y^IRS^*] for the *Y* coordinate, and [*X^GPS^*, *X^IRS^*] for the *X* coordinate. The approach phase was filtered for each landing. These 36 landings form a real data set.

The data were useful to carry out the learning and validation process for the predictive model creation and to determine decision thresholds *U* that were used to decide if there was an anomaly or not. There was a predictive model for each coordinate. Likewise, each sensor had its *U* threshold for each coordinate.

The algorithm NADCA-L was used for *X^GPS^*, *X^IRS^*, *Y^IRS^*, *Z^GPS^*, and *Z^IRS^*. The algorithm NADCA-O was used for *Y^GPS^*, *Y^ILS^*, *Z^ILS^*, and *Z^RA^* where *L* was created from the *SG* filter.

Section 4.1 shows some figures to visualize the sensor values of a real landing. These values are represented with the help of the runway coordinate system according to the *X*, *Y*, and *Z* axis.

### 4.1. Example of Real Landing

#### 4.1.1. Z Axis

**Figure 8:**
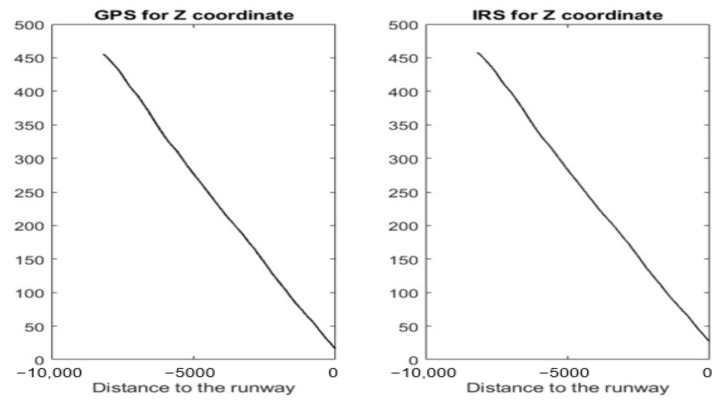
GPS and IRS for the *Z* coordinate (real values of a landing).

**Figure 9:**
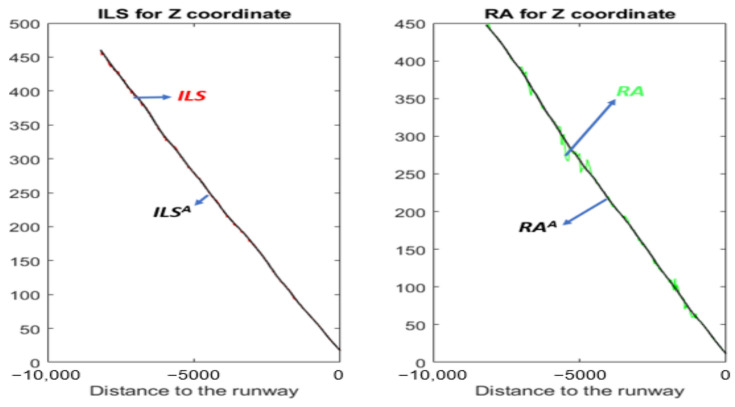
ILS, *ILS^L^*, RA, and *RA^L^* for the *Z* coordinate (real values of a landing).

Figure 8 and Figure 9 show the GPS, IRS, ILS, and RA values of a real landing according to the *Z* axis. In Figure 9, the *ILS^L^* and *RA^L^* values are represented by a black line. Those values are the corresponding smoothed signals of ILS and RA using the SG filter.

#### 4.1.2. Y Axis

**Figure 10:**
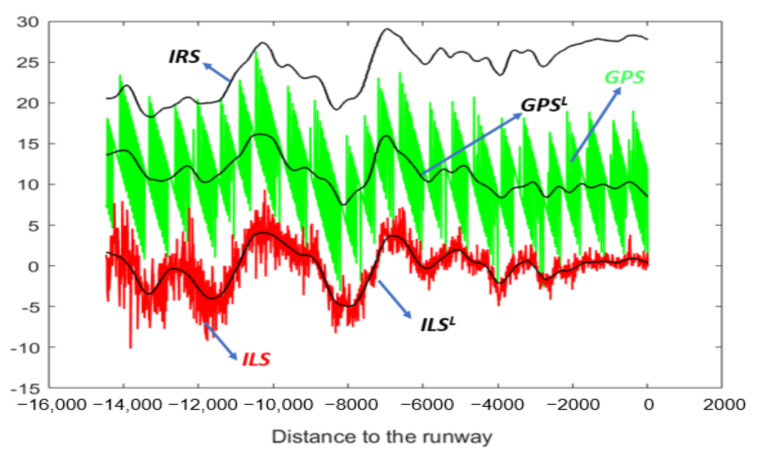
IRS, GPS, *GPS^L^*, ILS, and *ILS^L^* for the *Y* coordinate (real values of a landing).

Figure 10 shows the GPS, IRS, and ILS values of a real landing according to the *Y* axis. The GPS values are not exactly the expected values of a GPS sensor. Normally, a GPS sensor should give similar values to the *GPS^L^* signal. Consequently, a *GPS^L^* is required to process this pseudo-GPS (*P_GPS*) data. The *GPS^L^* and *ILS^L^* values are represented by a black line. Those values are the corresponding smoothed signals of *P_GPS* and ILS using the SG filter.

#### 4.1.3. X Axis

**Figure 11:**
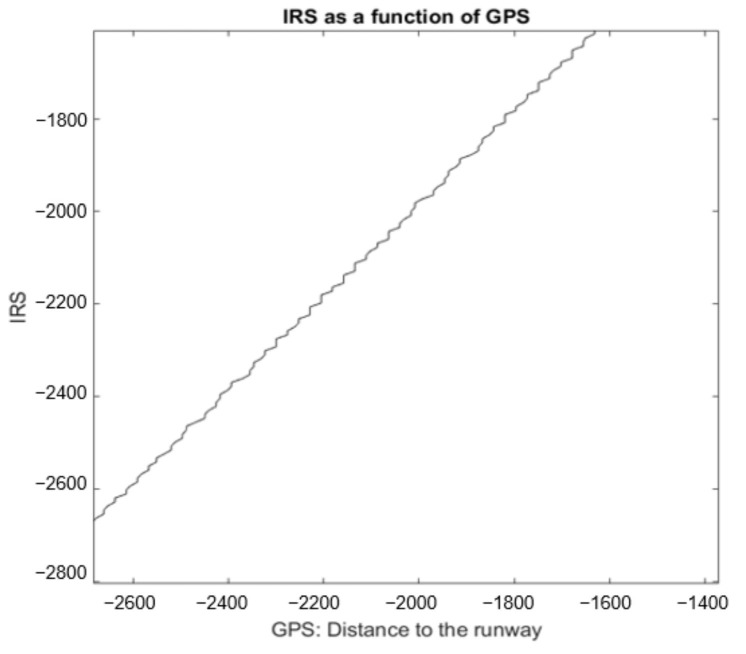
IRS portion as a function of GPS (real values for the *X* coordinate).

Figure 11 shows a portion of IRS values as a function of GPS values of a real landing according to the X axis. This portion is not a perfect line at a 45 degree angle. In general, this angle increases as the plane approaches the runway.

### 4.2. Predictive Model Using Real Landings

In this section, three predictive models (*PM^Z^*, *PM^Y^*, and *PM^X^*) for real data according to the *X*, *Y*, and *Z* axes are analyzed. Each predictive model only works with signals without oscillations. In this way, the convergence of the learning process is better and the anomaly detection process is more robust. On the other hand, data preparation is more laborious because signals with oscillations are smoothed using the SG filter.

Each predictive model was created using 30,554 examples for training and 15,050 examples for validation.

#### 4.2.1. Z Axis

**Figure 12:**
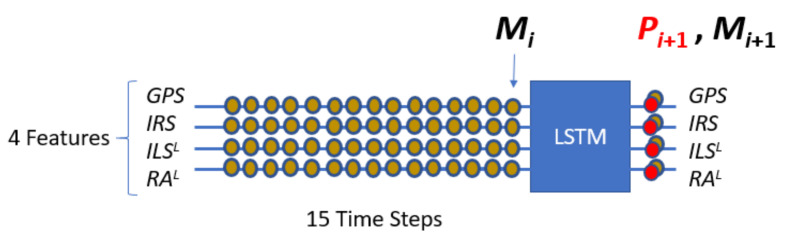
*PM^Z^* (real values).

Figure 12 represents *PM^Z^*. This model uses the data from GPS, IRS, *ILS^L^*, and *RA^L^*. *PM^Z^* is a stacked LSTM model. For clarity, the *Z* coordinate has been omitted in the figure.

Each example used to create *PM^Z^* contains *ND* + 1 consecutive differences where the last difference is the target that the model should predict from a set of *NM* previous measurements (*NM* = 15). This set of examples was split into two parts. This was a train-validation split. The first part was used to create the LSTM model. The remaining examples were used to evaluate the model.

The selected LSTM network architecture has three LSTM layers and 300 cells per layer. Using this architecture, the learning process adapts the weights of network. To do this, a backpropagation algorithm was used together with the set of learning examples. This algorithm, in addition to the number of layers and cells per layer, requires some hyperparameters to be defined. Specifically, the optimization algorithm (used to train the network) is Adam’s algorithm and the loss function (used to evaluate the network that is minimized by the optimization algorithm) is mean squared error (mse). The number of epochs (an epoch is one pass through all samples in the training dataset and updating the network weights) is 70. The batch size (a batch is one pass through a subset of samples in the training dataset after which the network weights are updated) is 32. The activation function is Relu (an activation is required to allow the neural network the ability to model non-linear processes).

The network can be trained using the learning examples and simultaneously, it can also be evaluated with the help of the validation examples. This evaluation provides an estimate of the performance of the network at making predictions for unseen data in the future.

**Figure 13:**
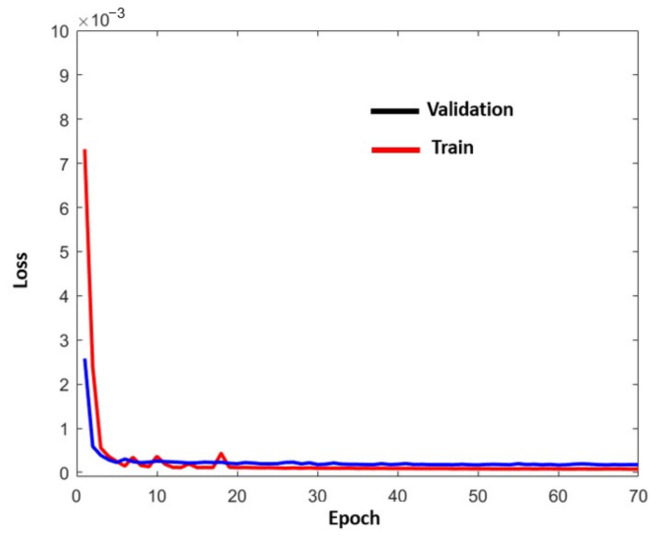
Evaluation curve for the *Z* coordinate using real landing values.

A positive evaluation means a good fit between the learning and validation sets. A good fit is a case where the performance of the model is good on both the training and validation sets. This can be evaluated from a plot (loss as a function of the number of epochs) where the train and validation losses decrease and stabilize around the same point. With this result, behaviors such as overfitting and underfitting are avoided. Figure 13 shows the training and validation loss meeting. The convergence of the curves is fast and stable. Similar results can be obtained using different sets of examples for a train-validation split.

#### 4.2.2. Y Axis

**Figure 14:**
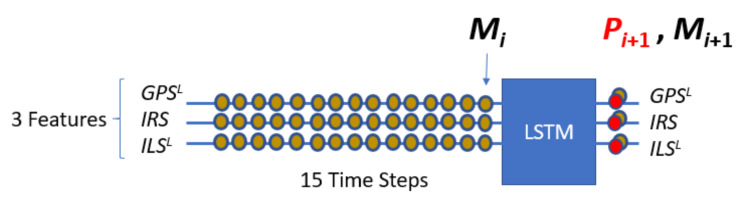
*PM^Y^* (real values).

Figure 14 represents *PM^Y^*. This model used the data from *GPS^L^*, IRS, and *ILS^L^*. For clarity, the *Y* coordinate has been omitted in the figure. *PM^Y^* is a stacked LSTM model. It has 3 layers of 300 cells each. The number of previous measurements is 15.

**Figure 15:**
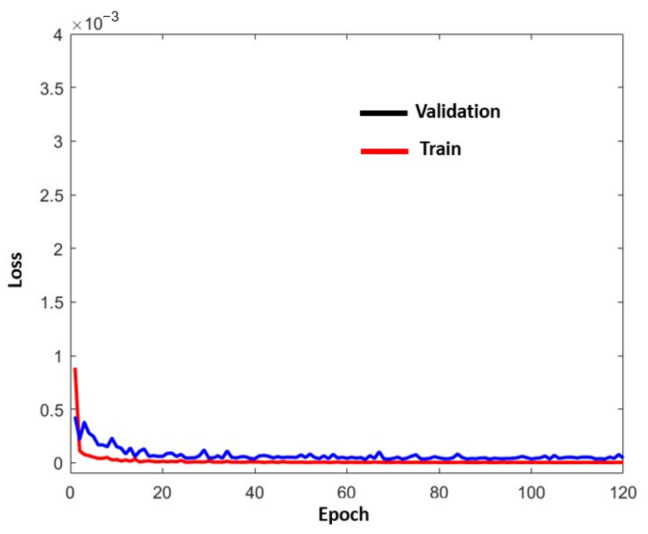
Evaluation curve for the *Y* coordinate using real landing values.

The convergence of the curves is fast and stable (see Figure 15).

#### 4.2.3. X Axis

**Figure 16:**
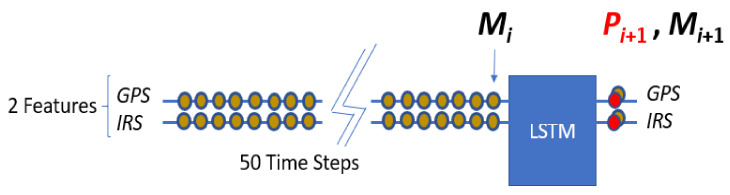
Predictive model for the *X* coordinate (real values).

Figure 16 represents *PM^X^*. This model uses the data from GPS and IRS. For clarity, the *X* coordinate is omitted in the figure.

**Figure 17:**
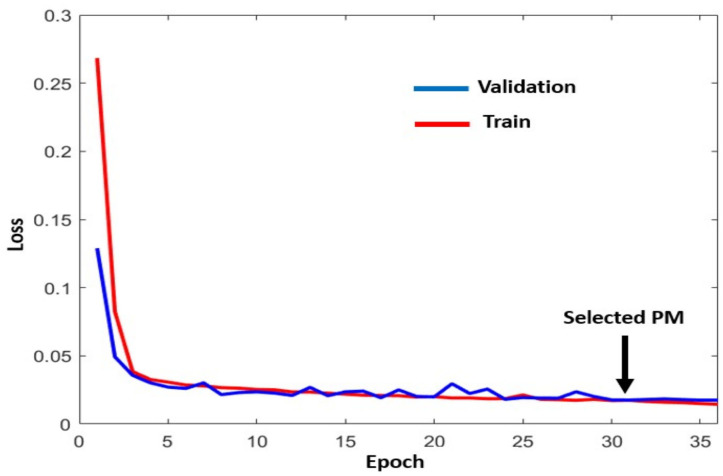
Evaluation curve for the *X* coordinate using real landing values.

*PM^X^* is a stacked LSTM model. It has 3 layers of 440 cells each. The number of previous measurements is 50. The number of previous measures as well as the number of cells per layer were increased to achieve a better fit between the learning and validation sets (see Figure 17).

The validation and learning graphs crossed and slightly diverged from epoch 32. From this epoch, overfitting appeared. To avoid this, the *PM^X^* for epoch 32 was selected.

This *PM^X^* is not the best possible model. This means that this model gives a prediction error greater than an optimal solution. A higher number of real landings (i.e., more examples) should prevent overfitting and provide a better *PM^X^*.

As discussed in Section 4.3.3, this *PM^X^* provided a prediction error acceptable for the IRS. However, the prediction error is important for GPS data. Consequently, this model was only used to detect anomalies in *X^IRS^*.

NADCA was primarily tested on the *Z* and *Y* axes because they are more diverse and contain more complicated signals than the *X* axis. The *X* axis only contains signals without oscillations. However, the *Z* and *Y* axes have signals with and without oscillations. In addition, the signals without oscillations have non-standard behavior.

### 4.3. Thresholding Using Real Landings

This subsection explains the *U* thresholds for each sensor and coordinate. *U* represents a prediction error when the time series does not show oscillations. *U* represents a maximum error for each sample between a smooth signal *L* and the corresponding raw values when the time series shows oscillations. Each threshold is denoted as $U_{Coordinate}^{Sensor}$.

#### 4.3.1. Z Axis

Prediction errors are calculated using *PM^Z^* and data without anomalies.

**Figure 18:**
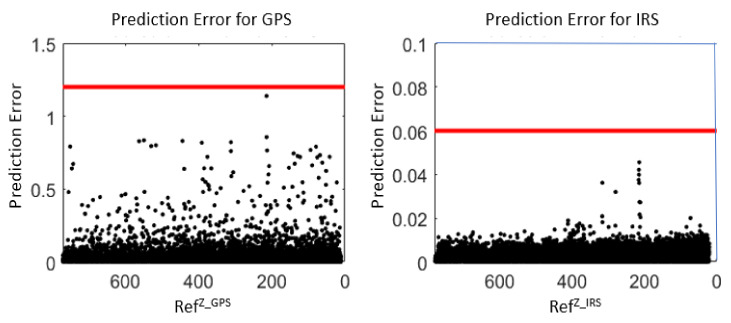
Envelopes for GPS and IRS using real values.

Figure 18 shows the prediction error for *Z^GPS^* and *Z^IRS^*. $Ref^{Z\_GPS}$ and $Ref^{Z\_GPS}$ represent $P_{i+1}^{Z\_GPS}$ and $P_{i+1}^{Z\_IRS}$ value sets (for the *Z* coordinate), respectively. These values are altitudes.

The *Z^IRS^* threshold can be set to $U_{Z}^{IRS}$ = 0.06. This result is good to detect anomalies. On the other hand, the *Z^GPS^* threshold can be set to $U_{Z}^{GPS}$ = 1.2. This threshold is also small and acceptable to detect anomalies. However, $U_{Z}^{GPS}$ is higher than $U_{Z}^{IRS}$. This means that *Z^GPS^* data may have minor anomalies.

**Figure 19:**
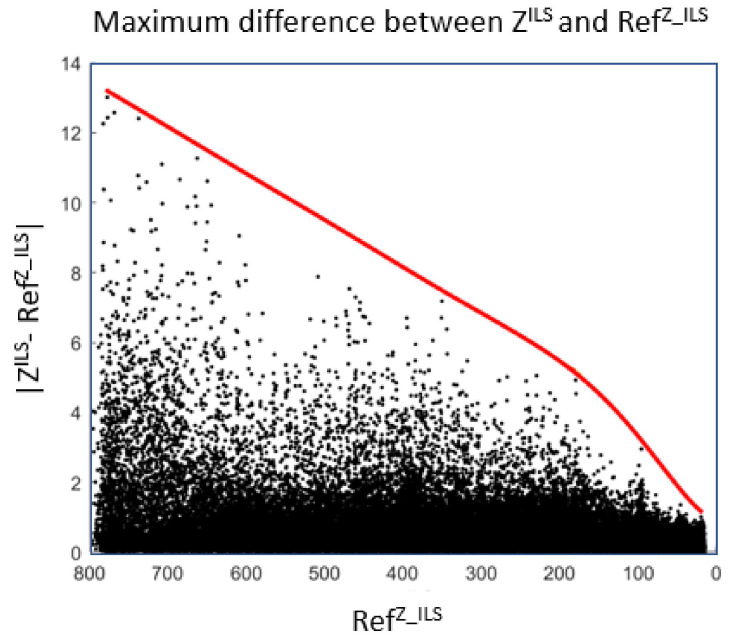
Envelope for ILS using real values.

For ILS, $U_{Z}^{ILS}$ is the envelope of the maximum error between $Ref^{Z\_ILS}$ and $Z^{ILS}$, where $Ref^{Z\_ILS}$ is the set of predicted values using ${Z^{ILS}}^{L}$ (see Figure 19).

**Figure 20:**
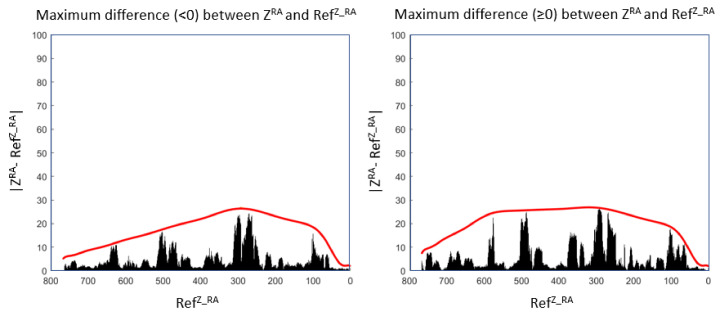
An envelope for the positive differences and another for the negative ones using real RA values.

For RA, $U_{Z}^{RA}$ is determined with the help of two envelopes, one envelope for positive differences and another for negative ones. Each envelope corresponds to the maximum error between $Ref^{Z\_RA}$ and $Z^{RA}$, where $Ref^{Z\_RA}$ is the set of predicted values using ${Z^{RA}}^{L}$ (see Figure 20).

#### 4.3.2. Y Axis

Prediction errors are calculated using *PM^Y^* and data without anomalies. The thresholds for *P_GPS* and IRS are a constant. $U_{Y}^{GPS}$ = 14 is the envelope of the maximum error between $Ref^{Y\_GPS}$ and $Y^{GPS}$ where $Ref^{Y\_GPS}$ is the set of predicted values using ${Y^{GPS}}^{L}$. *Y ^IRS^* is the only signal without oscillations. The maximum prediction error determines a threshold $U_{Y}^{GPS}$ = 0.35.

**Figure 21:**
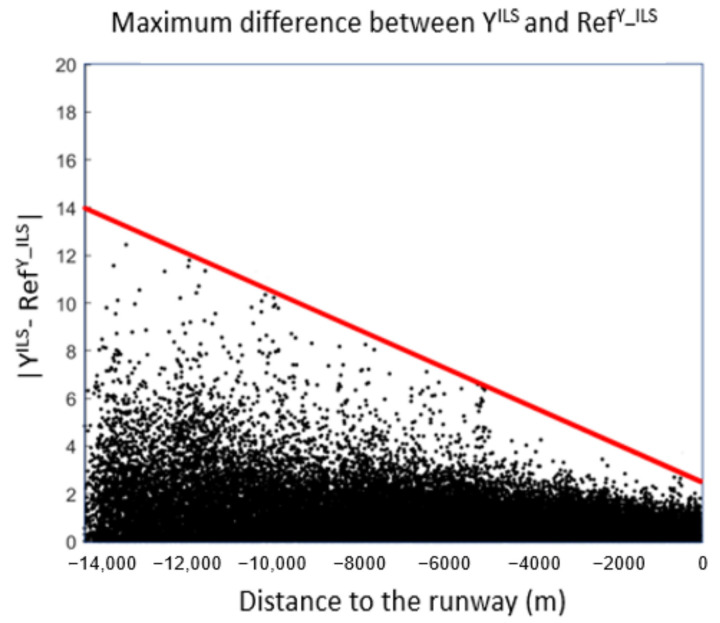
Envelope for ILS using real values for the *Y* coordinate.

For ILS, $U_{Y}^{ILS}$ is the envelope of the maximum error between $Ref^{Y\_ILS}$ and $Y^{ILS}$ where $Ref^{Y\_ILS}$ is the set of predicted values using ${Y^{ILS}}^{L}$ (see Figure 21).

#### 4.3.3. X Axis

Prediction errors are calculated using *PM^X^* and data without anomalies. The thresholds for GPS and IRS are a constant because these are signals without oscillations. The maximum prediction error for IRS determines a threshold $U_{X}^{IRS}$ = 0.35. It is good to detect anomalies. However, the maximum prediction error for GPS sets a threshold $U_{X}^{GPS}$ = 14, too high to detect anomalies. The chosen *PM^X^* is not the best possible model.

## 5. Examples of Anomaly Detection and Correction

In this section, real and simulated anomalies in real landing signals are detected and corrected using NADCA. For anomalies of long duration, Equation (7) was used. Section 5.1 explains how the parameter α of Equation (8) was determined.

### 5.1. Determination of the Parameter α

**Figure 22:**
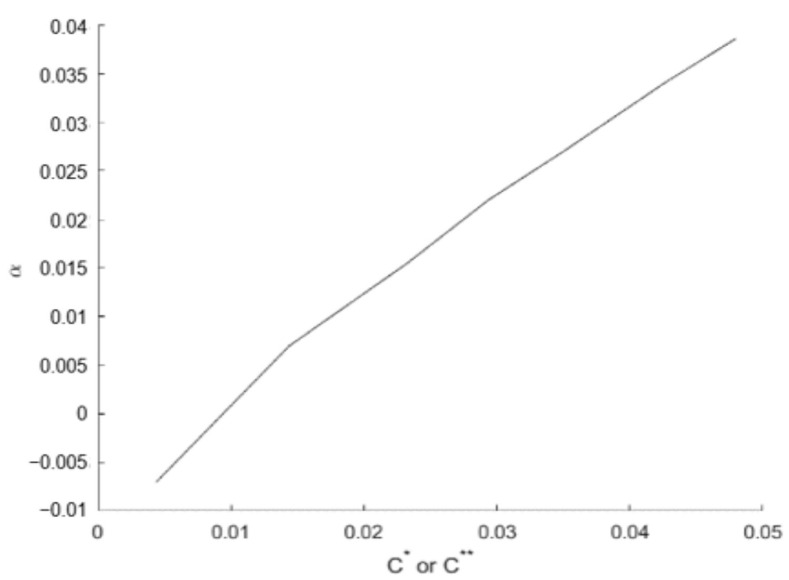
Relationship between α and $C^{*}$ (or $C^{**}$).

The parameter α of Equation (8) can be determined using a relationship between α and $C_{i}^{*}$. This relationship was found experimentally using a set of different examples with anomalies. For each example, the best α and its corresponding $C_{i}^{*}$ are selected. Figure 22 shows the result obtained for the GPS *Z*-coordinate.

### 5.2. Real Anomalies

This subsection presents two real anomalies that were detected and corrected by NADCA.

#### 5.2.1. Scale Factor Anomaly

**Figure 23:**
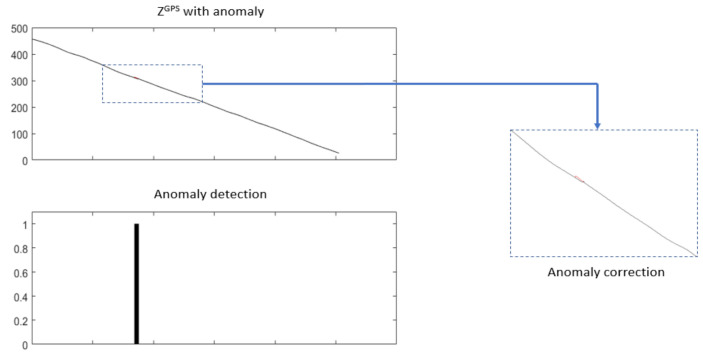
Anomaly detected and corrected using NADCA-L. The small anomaly appears in red.

This anomaly affected *Z^GPS^* values for one landing. It is a small scale factor anomaly that was detected and corrected using NADCA-L (see Figure 23).

#### 5.2.2. Noise Anomaly

**Figure 24:**
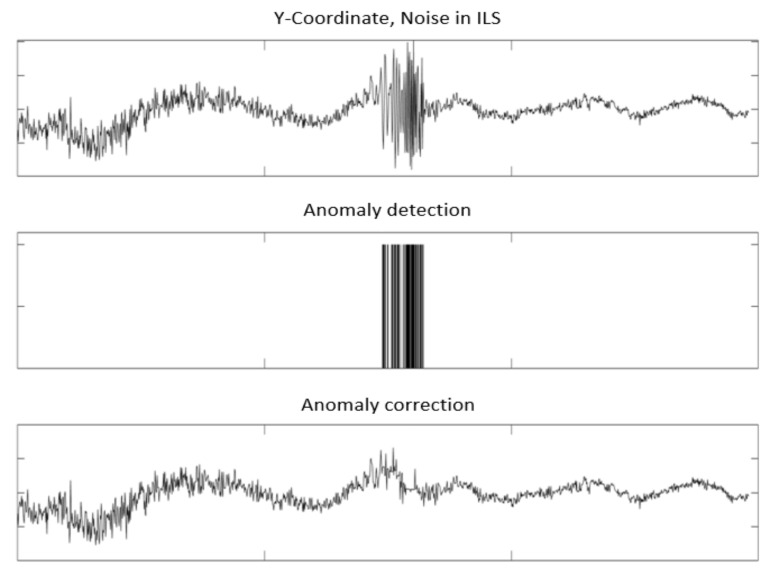
Anomaly detected and corrected using NADCA-O.

This anomaly appeared at *Y^ILS^*. It can be interpreted as noise. This anomaly was detected and corrected using NADCA-O (see Figure 24).

### 5.3. Simulated Anomalies

This subsection presents some simulated anomalies that appear in different landings. Unlike real anomalies, simulated anomalies are evaluated using two parameters: *F*-score [25] and root mean square error (RMSE) [26].

*F*-score compares the binary plot of the detected anomaly (DBP) and the “True” binary plot (TBP) that represents where the anomaly was generated. The value varies between 0 and 1. The best result is 1. It is useful to evaluate anomaly detection in a simple way by a number.

Assume that an anomaly appears in the time interval [*T*1, *T*2]. RMSE calculates the error between the original signal without anomaly and the signal with anomaly correction in the interval [*T*1, *T*2]. It is useful to evaluate anomaly correction, especially in signals without oscillations.

#### 5.3.1. Example 1: Landing with Bias in Z^GPS^ and Noise in Y^ILS^

**Figure 25:**
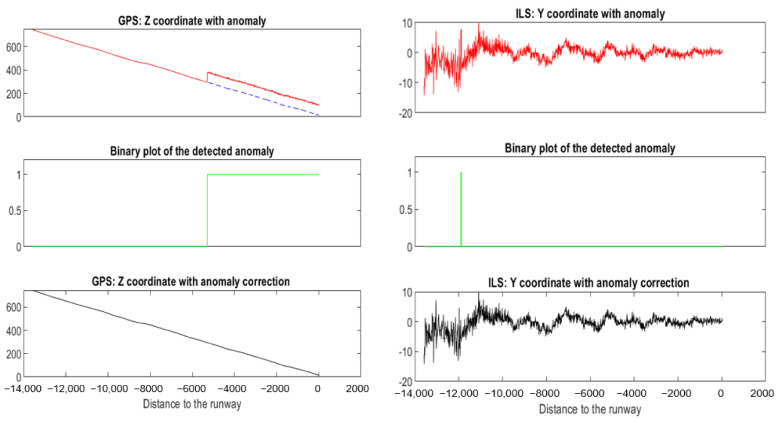
Bias and small noise anomalies detected and corrected on a specific landing using NADCA.

Figure 25 shows two anomalies on a specific landing. The bias anomaly in *Z^GPS^* is a simulated anomaly. The noise anomaly in *Y^ILS^* is a small real anomaly.

Table 1 shows the result for each signal of this landing using NADCA. There is a small anomaly in *Y^ILS^*. However, this anomaly was not artificially generated. Consequently, RMSE and *F*-Score calculation are not possible. There is an anomaly in *Z^GPS^*. This anomaly was artificially generated. The *F*-score is 1 because NADCA perfectly detects the anomaly. The RMSE is 0.57. This value is small. There are no anomalies in *X^GPS^*, *X^IRS^*, *Y^GPS^*, *Y^IRS^*, *Z^IRS^*, *Z^ILS^*, or *Z^RA^* and consequently, the value of *F*-score and RMSE is N/A.

#### 5.3.2. Example 2: Landing with Noise in Z^GPS^

**Figure 26:**
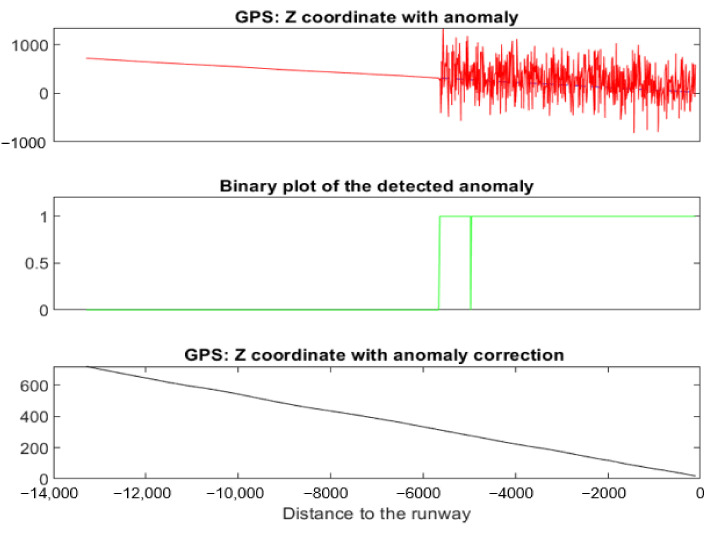
Noise anomaly detected and corrected on a specific landing using NADCA.

Figure 26 shows a simulated noise anomaly on *Z^GPS^*.

Table 2 shows the result for each signal of the landing using NADCA. There is an anomaly in *Z^GPS^*. The *F*-score (see Table 2) is 0.99 because TBP is determined prior to detection without discontinuities and DBP has a no anomalous sample anomaly. That sample intersects the NADCA correction. The binary plot of the detected anomaly shows that sample.

The RMSE is 0.52. This value is small. There are no anomalies in *X^GPS^*, *X^IRS^*, *Y^GP^*^S^, *Y^IRS^*, *Y^ILS^*, *Z^IRS^*, *Z^ILS^*, or *Z^RA^* and consequently, the value of *F*-score and RMSE is N/A.

#### 5.3.3. Example 3: Landing with a Noisy Bias in Z^GPS^

**Figure 27:**
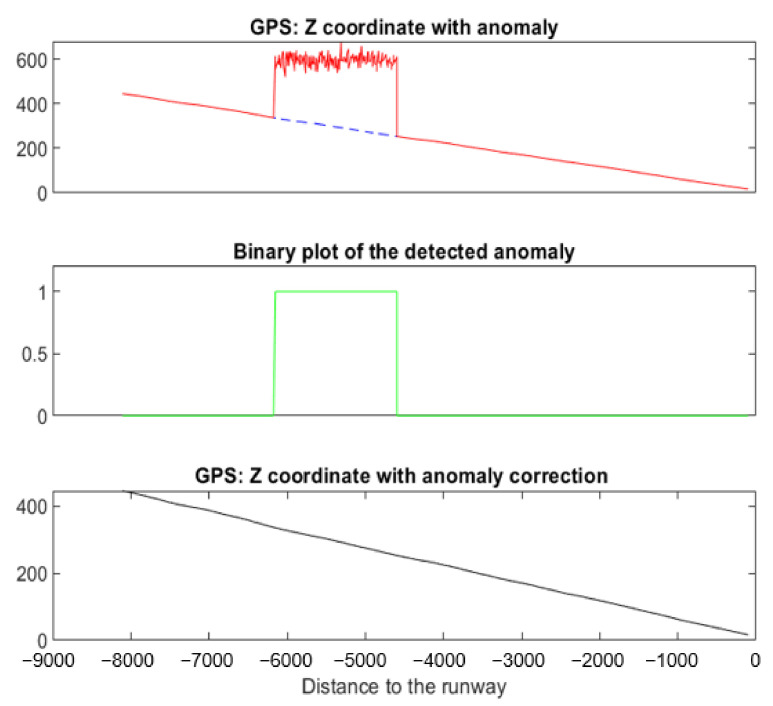
Noisy bias anomaly detected and corrected on a specific landing using NADCA.

Figure 27 shows an example of a simulated noisy bias anomaly on *Z^GPS^*. The F-score (see Table 3) is 1. In this example, the correction has to be precise in order to connect with the end of the anomaly.

#### 5.3.4. Example 4: Landing with Drift in Z^GPS^

**Figure 28:**
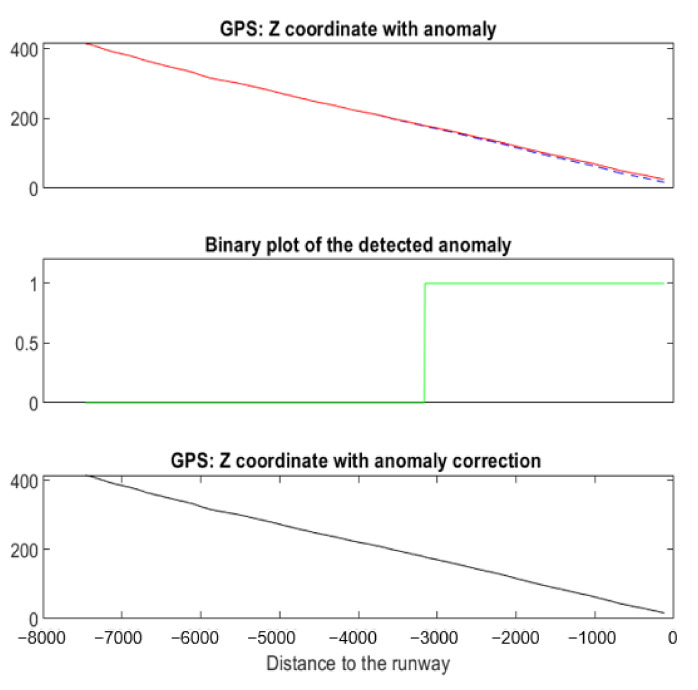
Drift anomaly detected and corrected on a specific landing using NADCA.

Figure 28 shows an example of a simulated drift anomaly on *Z^GPS^*. The F-score (see Table 4) is 0.87. This value is lower than 1 because the anomaly was detected 80 samples after the starting point of the anomaly. That is, the anomaly has a slow-moving variation and anomaly detection only occurs when Equation (7) is satisfied. The correction with a RMSE = 0.43 is of good quality.

#### 5.3.5. Example 5: Landing with Anomaly in Y^GPS^

**Figure 29:**
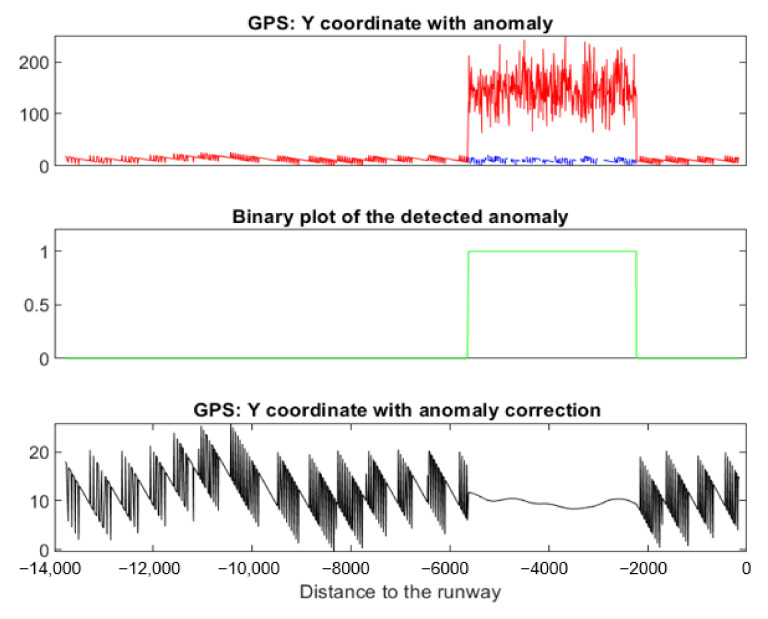
Drift anomaly detected and corrected on a specific landing using NADCA.

Figure 29 shows an example of a simulated noisy bias anomaly on *Y^GPS^*. The RMSE is 0.86 (see Table 5). The RMSE was calculated using the anomaly correction and the corresponding portion of the smoothed signal of the signal without anomaly. This calculation is different from the RMSE of a signal without oscillations. Thanks to the oscillations, other corrections are possible. Consequently, a higher RMSE value could also be an acceptable correction. The *F*-score is 1.

#### 5.3.6. Example 6: Coupling with Anomaly in X^IRS^

**Figure 30:**
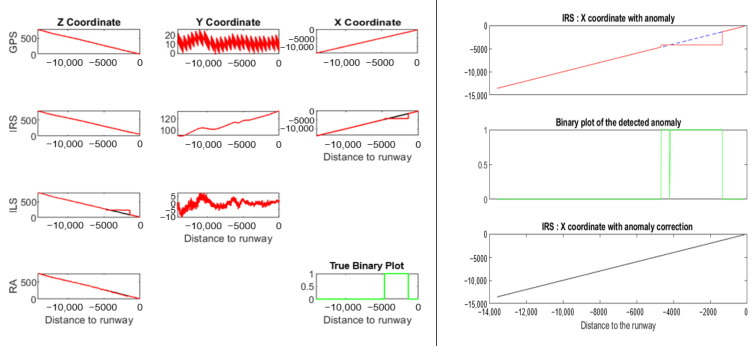
On the left side, coupling problem between *X^IRS^* and *Y^ILS^* for a drift anomaly on *X^IRS^*. On the right side, the anomaly detection and correction on *X^IRS^*.

Figure 30 shows, on the left side, a simple example of coupling between *X^IRS^* and *Y^ILS^* for a simulated anomaly in *X^IRS^*. The *Y^ILS^* values are calculated using Equation (2) where *X* = *X^IRS^*. A simulated anomaly appears in both *X^IRS^* and *Y^ILS^.* A small coupling between *X^IRS^* and *Z^RA^* is also present. The *H_terrain_* value of Equation (1) was obtained using *X^IRS^* values.

NADCA works following the order *X*, *Y*, and *Z.* It detects and corrects the anomaly in *X^IRS^* and consequently the anomaly does not appear in *Y^ILS^* and *Z^RA^*. If NADCA correctly detects the anomaly in *X^IRS^*, then there is no coupling problem and NADCA knows that the source of the anomaly is in *X^IRS^*. The right side of Figure 30 shows the anomaly detection and correction on *X^IRS^*.

NADCA can also work after each sample has been generated for each signal, even if there is a coupling problem. Anomalies in *X^IRS^*, *Y^ILS^*, and *Z^RA^* could be detected and corrected. However, the source of the anomaly would not be clear.

Table 6 shows a *F*-score of 0.99 due to a non-anomalous sample and a RMSE = 0.61.

#### 5.3.7. Example 7: Landing with Anomaly in Y^GPS^

**Figure 31:**
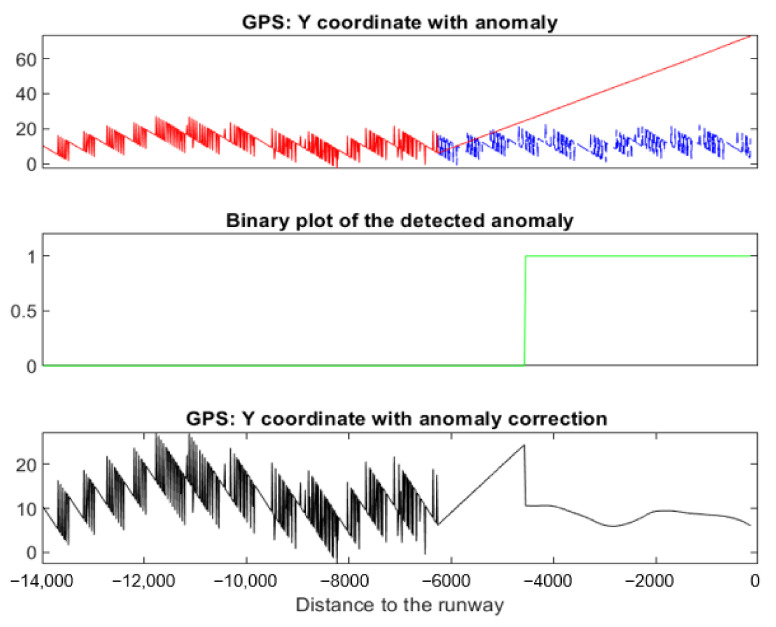
Drift anomaly detected and corrected on a specific landing using NADCA.

Figure 31 shows an example of a simulated drift anomaly on *Y^GPS^*. The RMSE is 2.9 (see Table 7). The RMSE was calculated using the anomaly correction and the corresponding portion of the smoothed signal of the signal without anomaly.

The *F*-score is 0.86. This value is not 1 because NADCA can only detect the anomaly when the anomalous values leave the zone of normal oscillations.

Equation (5) is not the only criterion used to start analyzing a possible slow-moving anomaly. For signals with oscillations, such as the *Y^GPS^*, consecutive raw data differences might be a better criterion than using the $C_{i}^{*}$ parameter.

#### 5.3.8. NADCA Overall Assessment

NADCA was evaluated using a set of 80 simulated sensor anomalies during landing. An average *F*-score value of 0.97 was obtained in relation to the detection of anomalies and an average root mean square error (RMSE) value of 2.10 regarding the correction of anomalies.

The average F-score value is very high. It does not reach the value 1 because, mainly, NADCA consumes some samples before detecting slow moving anomalies. The average RMSE value is acceptable. This could be lower considering, for example, a higher ND number (see Section 3 where ND = 14). However, a low ND is preferable. In this way, NADCA can start working as soon as possible. This is important since there are landings that do not last a long time.

Other strategies for correction could have been considered, for example, using algorithms described in [27]. However, preference has been given to using the same prediction algorithm that simultaneously allows both detecting and correcting anomalies with acceptable quality.

## 6. Discussion

NADCA is an algorithm for the detection and correction of anomalies in time series. The algorithm differentiates between time series with oscillations and without oscillations.

Three versions of NADCA have been described. NADCA-B is only useful for detecting some obvious anomalies, NADCA-L detects and corrects anomalies in signals without oscillations, and NADCA-O detects and corrects anomalies in signals with oscillations. NADCA-B can be seen as a particular case of NADCA-L. Furthermore, NADCA-L is a special case of NADCA-O.

NADCA is robust because the predictions are made on smoothed signals. When a time series has oscillations, the algorithm creates a smooth signal by using the SG filter. A smoothed signal guarantees a small prediction error less than a constant.

NADCA has been used for both simulated and real anomalies on real landings.

NADCA is applied following the order of the coordinates *X*, *Y*, and *Z*. In this way, if an anomaly appears in any sensor coordinate, it is corrected before analyzing the next coordinate since the latter can be a function of the previous coordinate. Consequently, coupling problems are avoided.

Regarding the thresholds that derive from a prediction error, we can compare $U_{Z}^{GPS}=1.2$ and $U_{Z}^{IRS}=0.06$. One would expect them to be similar, which is not the case. This may originate from some samples in *Z^GPS^* that could be small anomalies. However, they may not be relevant.

The predictive model for the *X* axis is not the best to predict the behavior of *X^GPS^*. This comes from the fact that the model only combines two sensors and the number of landings used to create the model is small. On the other hand, for the *Y* and *Z* axes, despite the small number of landings, the models generalize well for the selected airport. This is so because each model uses more sensors in a compact way.

NADCA was developed primarily to detect and correct anomalies during the landing phase. During this phase, the plane does not make abrupt changes and therefore, NADCA detects anomalies related to the sensors’ operation. However, an abrupt change in the trajectory of the aircraft would generate changes in the sensor signals that would be considered anomalous. These changes usually happen during the approximation phase that has not been considered in this work.

It is uncertain whether each predictive model could correctly predict the behavior of the sensors for landings in another airport. This does not have to be the case, and therefore, it is left for future work to consider new landing data from various airports in order to create a predictive model that generalizes to any airport.

## 7. Conclusions

NADCA is a new algorithm for anomaly detection and correction in time series. The algorithm is robust because it differentiates between oscillating and non-oscillating time series and always makes predictions on smooth signals.

NADCA uses a predictive model based on an LSTM neural architecture. The predictions provide a reference. The difference between this reference and the raw values is compared with a specific threshold *U* to decide whether or not there is an anomaly. NADCA was tested in time series that describe the landing phase of an airplane with promising results. This algorithm guarantees the quality of measures during landing. Generalization to several airports could be considered if additional data sets from various airports were made available. Importantly, NADCA is a general-purpose algorithm that could also be used in other contexts. Future work will consider applying NADCA for applications in other domains.

The following points summarize the main conclusions of this paper:

1. NADCA is a new algorithm for anomaly detection and correction. Detection and correction are performed simultaneously.
2. NADCA uses a new prediction strategy to detect and correct both fast-changing and slow-moving anomalies.
3. NADCA distinguishes between signals with oscillations and without oscillations. The algorithm is similar for both types of signals, however, signals with oscillations require an additional step. This step consists of obtaining a smoothed signal in real time.
4. NADCA works in real time. It uses information from sensors in a compact way and only needs to predict one sample at each iteration.
5. NADCA evaluation has given an average F-score value of 0.97 for detection and an average RMSE value of 2.1 for correction.
6. The different examples in this article show the simultaneous detection and correction of both fast changing anomalies (e.g., Figure 27) and slow-moving anomalies (e.g., Figure 28). NADCA can deal with simultaneous anomalies in different sensors (e.g., Figure 25). Figure 30 shows how NADCA avoids the coupling problem.
7. Once the anomaly is detected, the corresponding sensor does not need to be isolated.

## Figures and Tables

**Table 1:** Result for each signal after using NADCA.

	*X^GPS^*	*X^IRS^*	*Y^GPS^*	*Y^IRS^*	*Y^ILS^*	*Z^GPS^*	*Z^IRS^*	*Z^ILS^*	*Z^RA^*
Anomaly	No	No	No	No	Yes	Yes	No	No	No
F-score	N/A	N/A	N/A	N/A	N/A	1	N/A	N/A	N/A
RMSE	N/A	N/A	N/A	N/A	N/A	0.57	N/A	N/A	N/A

**Table 2:** Result for each signal after using NADCA.

	*X^GPS^*	*X^IRS^*	*Y^GPS^*	*Y^IRS^*	*Y^ILS^*	*Z^GPS^*	*Z^IRS^*	*Z^ILS^*	*Z^RA^*
Anomaly	No	No	No	No	No	Yes	No	No	No
F-score	N/A	N/A	N/A	N/A	N/A	0.99	N/A	N/A	N/A
RMSE	N/A	N/A	N/A	N/A	N/A	0.52	N/A	N/A	N/A

**Table 3:** Result for each signal after using NADCA.

	*X^GPS^*	*X^IRS^*	*Y^GPS^*	*Y^IRS^*	*Y^ILS^*	*Z^GPS^*	*Z^IRS^*	*Z^ILS^*	*Z^RA^*
Anomaly	No	No	No	No	No	Yes	No	No	No
F-score	N/A	N/A	N/A	N/A	N/A	0.99	N/A	N/A	N/A
RMSE	N/A	N/A	N/A	N/A	N/A	0.43	N/A	N/A	N/A

**Table 4:** Result for each signal after using NADCA.

	*X^GPS^*	*X^IRS^*	*Y^GPS^*	*Y^IRS^*	*Y^ILS^*	*Z^GPS^*	*Z^IRS^*	*Z^ILS^*	*Z^RA^*
Anomaly	No	No	No	No	No	**Yes**	No	No	No
F-score	N/A	N/A	N/A	N/A	N/A	**0.87**	N/A	N/A	N/A
RMSE	N/A	N/A	N/A	N/A	N/A	**0.43**	N/A	N/A	N/A

**Table 5:** Result for each signal after using NADCA.

	*X^GPS^*	*X^IRS^*	*Y^GPS^*	*Y^IRS^*	*Y^ILS^*	*Z^GPS^*	*Z^IRS^*	*Z^ILS^*	*Z^RA^*
Anomaly	No	No	Yes	No	No	No	No	No	No
F-score	N/A	N/A	1	N/A	N/A	N/A	N/A	N/A	N/A
RMSE	N/A	N/A	0.86	N/A	N/A	N/A	N/A	N/A	N/A

**Table 6:** Result for each signal after using NADCA.

	*X^GPS^*	*X^IRS^*	*Y^GPS^*	*Y^IRS^*	*Y^ILS^*	*Z^GPS^*	*Z^IRS^*	*Z^ILS^*	*Z^RA^*
Anomaly	No	Yes	No	No	No	No	No	No	No
F-score	N/A	0.99	N/A	N/A	N/A	N/A	N/A	N/A	N/A
RMSE	N/A	0.61	N/A	N/A	N/A	N/A	N/A	N/A	N/A

**Table 7:** Result for each signal after using NADCA.

	*X^GPS^*	*X^IRS^*	*Y^GPS^*	*Y^IRS^*	*Y^ILS^*	*Z^GPS^*	*Z^IRS^*	*Z^ILS^*	*Z^RA^*
Anomaly	No	No	Yes	No	No	No	No	No	No
F-score	N/A	N/A	0.84	N/A	N/A	N/A	N/A	N/A	N/A
RMSE	N/A	N/A	2.9	N/A	N/A	N/A	N/A	N/A	N/A
